# Unfathomed Nanomessages to the Heart: Translational Implications of Stem Cell-Derived, Progenitor Cell Exosomes in Cardiac Repair and Regeneration

**DOI:** 10.3390/cells10071811

**Published:** 2021-07-17

**Authors:** Charan Thej, Raj Kishore

**Affiliations:** 1 Center for Translational Medicine, Lewis Katz School of Medicine, Temple University, Philadelphia, PA 19140, USA; Charan.thej.gurrala@temple.edu; 2 Department of Cardiovascular Sciences, Lewis Katz School of Medicine, Temple University, Philadelphia, PA 19140, USA

**Keywords:** stem cells, progenitor cells, exosomes, cardiovascular disease, cardiac repair

## Abstract

Exosomes formed from the endosomal membranes at the lipid microdomains of multivesicular bodies (MVBs) have become crucial structures responsible for cell communication. This paracrine communication system between a myriad of cell types is essential for maintaining homeostasis and influencing various biological functions in immune, vasculogenic, and regenerative cell types in multiple organs in the body, including, but not limited to, cardiac cells and tissues. Characteristically, exosomes are identifiable by common proteins that participate in their biogenesis; however, many different proteins, mRNA, miRNAs, and lipids, have been identified that mediate intercellular communication and elicit multiple functions in other target cells. Although our understanding of exosomes is still limited, the last decade has seen a steep surge in translational studies involving the treatment of cardiovascular diseases with cell-free exosome fractions from cardiomyocytes (CMs), cardiosphere-derived cells (CDCs), endothelial cells (ECs), mesenchymal stromal cells (MSCs), or their combinations. However, most primary cells are difficult to culture in vitro and to generate sufficient exosomes to treat cardiac ischemia or promote cardiac regeneration effectively. Pluripotent stem cells (PSCs) offer the possibility of an unlimited supply of either committed or terminally differentiated cells and their exosomes for treating cardiovascular diseases (CVDs). This review discusses the promising prospects of treating CVDs using exosomes from cardiac progenitor cells (CPCs), endothelial progenitor cells (EPCs), MSCs, and cardiac fibroblasts derived from PSCs.

## 1. Introduction

Cardiovascular diseases (CVDs) such as coronary heart disease, heart failure, atherosclerosis, congenital heart disease, arrhythmic disorders, coronary artery disease, and peripheral artery disease are still the primary cause of health burden globally [1]. Today, ischemic heart disease remains the leading cause of death worldwide (https://www.who.int/news-room/fact-sheets/detail/the-top-10-causes-of-death; accessed on 25 June 2021). The global prevalence of CVD was 485.6 million in 2017, which was 28% higher than in 2007, and about 18 million deaths were associated with CVD in 2017, 21.1% higher than the deaths recorded in 2007 [2]. Tobacco use, physical inactivity, poor nutrition, obesity, high blood cholesterol, high blood pressure, and diabetes remain the key factors that cause CVDs. The administration of statins, β-blockers, angiotensin-converting enzyme (ACE) inhibitors, angiotensin II-receptor blockers (ARBs), or non-steroidal anti-inflammatory drugs such as aspirin is the current standard of care (SOC) for the clinical management of several CVDs [3]. Current surgical SOC procedures such as angioplasty, cardiomyoplasty, atherectomy, coronary artery bypass graft surgery (CABG), percutaneous coronary intervention (PCI), and heart transplantation are all invasive (https://www.heart.org/en/health-topics/heart-attack/treatment-of-a-heart-attack/cardiac-procedures-and-surgeries; accessed on 25 June 2021) [4].

There is an urgent need to develop minimally invasive and effective therapies for the clinical management of CVDs. Stem cell therapy has been widely explored for the treatment of CVDs over the last two decades. It is now well accepted that the mechanism of action (MoA) by which stem cells elicit regenerative responses is in a paracrine manner rather than by engraftment into the damaged tissues and cell replacement. Stem cells are known to secrete growth factors and cytokines that induce immunomodulation, angiogenesis, cell survival, and proliferation during homeostasis and injury. In addition to circulating growth factors and cytokines, growing evidence suggests that paracrine cell–cell communications are also mediated by extracellular vesicles (EVs), particularly ectosomes and exosomes, to prompt various cellular functions [5]. Most exosomes comprise surface transmembrane proteins such as CD9, CD63, CD81, integrins, and MHC complexes, which facilitate direct binding to target cell types. For example, MHC-I, MHC-II, CD80, and CD34 on umbilical cord blood dendritic cell exosome membranes can directly dock onto T cells and trigger a downstream cascade to promote the proliferation of T cells and further induce antitumor activity [6]. In addition, exosomes have been shown to fuse with the plasma membrane of target cells mediated by lipid rafts, integrins, and proteins such as Rab and SNAREs to deliver their cargo into target cells [7,8]. Exosomes are mostly endocytosed by lipid-raft-mediated endocytosis, phagocytosis, or micropinocytosis and fuse with the limiting membrane of the endocytic compartment to release their cargo [5,9].

For instance, a study showed that hypoxic MSC exosomes become endocytosed into cardiomyocytes (CMs) and promote a reduction in apoptotic markers cleaved caspase-3, bad, bax, and bcl-2 mediated by miR-210. Inhibition of exosome generation from hypoxic MSCs results in increased apoptosis of CMs, demonstrating that exosomal cell–cell communication is essential. Sahoo et al. [10] showed that conditioned medium derived from human CD34^+^ hematopoietic stem cells promoted long-lasting angiogenesis of HUVECs in vitro [10]. The depletion of exosomes in the conditioned medium resulted in a dramatic reduction of HUVEC tube formation, demonstrating the pivotal role of exosomes in promoting intercellular therapeutic functions. In vivo, these exosomes further lead to neovascularization in Matrigel plug and corneal angiogenesis assays [10]. The study showed that exosomes from CD34^+^ hematopoietic stem cells induced efficient angiogenesis compared to exosomes from mononuclear cells, implying that the stemness of cells could bring about enhanced therapeutic efficacy [10,11]. It has been demonstrated in the past that exosomes from embryonic and induced pluripotent stem cells (PSCs) enhance cardiac function and the angiogenesis, survival, and proliferation of cardiac progenitor cells (CPCs) in the ischemic myocardium of mice [12,13,14]. Other studies have also shown the efficacy of EVs or exosomes from CMs or cardiac progenitor cells (CPCs) derived from PSCs [15,16]. Although exosomes from PSCs, or PSC-derived CMs or CPCs, have shown promising results in promoting cardiac repair, complete regeneration has not been achieved so far. To further improve exosome-based therapeutic interventions for CVDs, there may be a need to restrategize. Due to the varied cellular composition of the myocardium, exosomes from a single cell source are unlikely to promote the complete repair and regeneration of the damaged heart. This review discusses the possibility of using a combination of exosomes from different progenitor cells such as endothelial progenitor cells (EPCs), mesenchymal stromal cells (MSCs), CPCs, and cardiac fibroblasts, which can be derived from the same parent embryonic or induced PSCs. To the best of our knowledge, this is the first review to elaborately discuss the necessity of using a combination of exosomes from an unlimited source of stem cells. The biogenesis, composition, and functions of different EVs, especially exosomes, are described in the next section of this review.

## 2. Biology of Exosomes

### 2.1. Biogenesis of Extracellular Vesicles, Microvesicles, and Exosomes

EVs are known to be released by almost every healthy or dying cell in the body. Beyond secreted proteins, small RNAs, and growth factors, exosomes are now recognized to be critical mediators of cell–cell communication during homeostasis and pathophysiological conditions, although the signaling mechanisms of endogenous exosomes in tissue homeostasis remain to be established. Their differential biogenesis is broadly classified as apoptotic bodies, microvesicles/microparticles, and exosomes. In apoptotic cells, caspase 8 and 9 cascade activation induces nuclear chromatin condensation, followed by splitting, forming micronuclei, leading to blebbing and the separation of apoptotic bodies or apoptosomes [17]. These apoptotic bodies can range from 500 nm to 2μm in diameter [17].

On the other hand, microparticles or microvesicles are formed by the budding of the plasma membrane and are 100 nm to 1000 nm in diameter [18]. Both apoptotic bodies and microparticles carry surface and intracytoplasmic antigens of the parent cells; however, they can be distinguished based on size and the DNA fragments carried by apoptotic cells. Thus, both apoptotic bodies and most microvesicles can be separated ideally by 16,000–20,000× *g* sedimentation, microfluidics-based separation, or size-exclusion chromatography [18].

In 1983, two studies published by separate scientific groups within a week demonstrated the formation and release of microvesicles for the first time while studying transferrin receptor recycling in sheep and rat reticulocytes [19,20]. Harding et al. directly visualized the formation of ~50 nm-sized vesicles using colloidal gold conjugation of the transferrin receptor and confirming that these particles result from endocytosis of the late endosome [20]. In contrast, Pan and Johnstone described a vesicle-shedding mechanism, and these microvesicles were later called ectosomes or microvesicles. Moreover, these MVs were not directly visualized at that time [19]. The term “exosome” was later coined by Rose Johnstone. These studies and many subsequent ones have demonstrated that, unlike other EVs, exosomes originate via the endocytic route due to the inward budding of the limiting membrane of endocytic compartments that fuse and lead to early endosome formation. The early endosomes internalize intracellular components by inward budding, initiated by the endosomal sorting complex required for transport (ESCRT) and tightly regulated by tetraspanins, ALIX, phospholipase D2, syntenin, and other molecules to further mature into multivesicular bodies (MVBs). The MVBs subsequently adhere to the plasma membrane and release the exosomes via exocytosis into the extracellular space [5]. Although there are a few reports that delineate the cardioprotective or reparative role of exosomal miRNAs, proteins, lipids, and other molecules, these exosomes or EV components are yet unfathomed. In the following two sections of this review, we discuss the exosomal cargo of cells in general and specific to the cargo of pluripotent stem cells (PSCs) and cardiac tissue-specific progenitors derived from them.

### 2.2. Exosomal Genetic Cargo

The genetic cargo composition of exosomes is mainly RNA [21], shorter noncoding oligonucleotides such as miRNA [12], circRNA [22], lncRNA [23], tRNA [24], piRNA [25], and scaRNA [26], which are known to mediate cell to cell communication in different organs and tissues in the body. A total of 343 chromosomal DNA sequences were detected in the exosomes/cardiosomes derived from adult mouse CMs a decade ago [27]. Although they demonstrated that chromosomal DNAs embedded within cardiosomes are transferred into target fibroblasts, this relocated DNA’s functions were not well elucidated. However, the same group showed that mRNAs from cardiosomes elicited metabolic and cellular processes in the target fibroblasts [27]. The RNA content of exosomes derived from different cell types is diverse. Their diversity is broadly classified as functionally translatable, nonfunctional, or based on their post-transcriptional roles. Although the RNA contents of exosomes derived from particular cells are partially similar, RNA profiling from these exosomes suggests that they significantly differ from their parent cell compositions and offer selective and specific RNA enrichment [28]. A class of long noncoding RNAs resulting from back splicing in the lariat splicing event process entrapped in exosomes could play a prominent role in CVD remodeling [22]. Experimental evidence indicates that extracellular vesicles (EVs) from human pulmonary aortic smooth muscle cells (HPASMCs) carry mRNA into human pulmonary aortic endothelial cells (HPAECs) and allow the translation of the mRNA cargo [29], suggestive of therapeutic implications in cardiovascular biology. The majority of RNAs are transported from the nucleus to the cytoplasm, together as a bigger ribonucleoprotein particle or independently, in association with RNA-binding proteins (RBPs) [30]. RNA biogenesis and subsequent selective enrichment in particular multivesicular bodies (MVBs) and then in exosomes seem to be mediated by post-translational modifications such as phosphorylation, ubiquitination, and SUMOylation [31].

Transcriptomic analysis of EVs derived from endothelial progenitor cells (EPCs), cardiac progenitor cells (CPCs), and cortical bone stem cells revealed the differential and similar composition of various mRNAs, miRNAs, and other noncoding RNAs [32]. Interestingly, this composition differed when parent cells were exposed to hyperglycemic conditions, implying selective genetic compositions based on the microenvironment or disease condition [32]. A class of long noncoding RNAs resulting from back splicing in the lariat splicing event process entrapped in exosomes could play a prominent role in CVD remodeling [22]. Recently, Garikipati VNS et al. demonstrated that circular RNA circFnd3b reduces cardiomyocyte apoptosis, promotes angiogenesis, and improves left ventricular functions when overexpressed in mouse hearts postmyocardial infarction (post-MI) [33]. Moreover, sequencing human serum exosomes has revealed an abundance of circular RNAs within them [34]. A recent study demonstrated that swine cardiosphere-derived cells (CDCs) improved cardiac functions in a swine model of dilated cardiomyopathy (DCM), and this functional improvement was found to be associated with their miRNA composition [35]. Several miRNAs such as miR-126-5p, miR-132-3p, miR-146a-5p, miR-181b, miR-210-3p, and miR-451a, known to induce antifibrotic, antiapoptotic, and proangiogenic properties, were found to be enriched in CDC exosomes from both human and swine compared to human cardiac fibroblast (HCF) exosomes. Pretreatment of CDCs with exosome inhibitor GW4869 abrogated the cardiac-protective functions of CDCs [35], confirming the role of their exosomes. The inhibition of specific pro-inflammatory and antiangiogenic miRNAs such miRNA 375 and miRNA 377 in bone marrow progenitor cells likely restricts the transfer of these miRNAs into the progenitor cell exosomes. When injected into MI-induced mice hearts, these cells release exosomes deficient in the targeted miRNAs, resulting in decreased inflammation and enhanced angiogenesis [36,37]. EPC exosomes from an IL-10 knockout mouse were enriched with integrin-linked kinase (ILK) and were deficient in myocardial repair after MI [38]. In contrast, wild-type EPC exosomes and EPC exosomes with downregulated ILK efficiently promoted cardiac protection, further confirming the pivotal role of exosomes and their cargo in disease, tissue repair, and regeneration [38]. A different study showed that miRNA-19a mediated phosphatase and tensin homolog (PTEN) suppression in endometrial MSC exosomes, further phosphorylating AKT and ERK to exert proangiogenic cardioprotective effects in ischemic rat hearts [39]. The miRNA-210-Efna3 pathway promoted by bone marrow MSC exosomes presented similar results in the rat MI model [40] In addition to miRNAs, the biogenesis of exosomes carrying different nucleic acids requires a sequential role of proteins and lipids in the endosomal pathway, including RABs, ALIX, TSG101, CD9, CD63, CD81, flotillin, and several kinds of lipids. The role of these exosome-associated proteins is discussed in the next section.

### 2.3. Exosomal Proteins and Lipids

A diverse range of proteins are known to be present within and on the surface of EVs. However, several proteomic analyses of various EV subpopulations revealed commonly expressed proteins in smaller EVs or exosomes. Proteomic analysis revealed markers such as heat shock protein-70 KDa (HSP-70/HSP-73, HSP-70/HSP-72), flotillin, and major histocompatibility complex present in larger EVs rather than only on exosomes [41]. The same study demonstrated that CD9, CD63, and CD81 are reliable markers in identifying exosomes originating via the endocytic pathway. ALIX and TSG101, driven by the ESCRT machinery, were associated with all types of EVs [41]. Several studies have demonstrated that serum exosome proteins differ from proteins carried by several cancer cells, and the detection of cancer-specific protein markers could help in reliable cancer diagnosis. For example, the detection of Glypican-1 in the exosomes collected from serum samples is likely to indicate the early stages of pancreatic cancer [42]. Some regenerative secreted proteins of the parent cells are mirrored in their exosomes. Angiogenic proteins such as vascular endothelial growth factor (VEGF), VEGF receptors, angiogenin, monocyte chemoattractant protein 1 (MCP-1), and urokinase-type plasminogen activator (UPAR) enriched in MSC EVs compared to their parent cells are functionally active and induce angiogenic functions [43]. Proteomic profiling of iPSC EVs revealed several proteins associated with cellular components, molecular function, and biological processes similar to those of their parent cells [13].

In addition to proteins, lipids on exosomes play a role in the transportation or internalization of exosomes into target cells. Protein–lipid interactions involved in the ESCRT machinery in the endosomal pathway are essential for the formation of exosomes. Phosphatidylserine and phosphatidylcholine on exosomes’ surface bind to mucin-like receptors or G-protein-coupled receptors (GPCR) on target cells, allowing the prediction of exosomal fate [44]. Exosomes directly transport several lipids such as eicosanoids, cholesterol, and fatty acids into recipient cells, playing a direct role in homeostasis or diseases. Exosomes from blood collected in a postprandial state expressed higher levels of CD36 and promoted CD36-mediated lipid uptake in cardiomyocytes and cardiac endothelial cells [45]. Previously, CD36 was found to be required for the uptake of long-chain fatty acid into the human myocardium, causing lipid accumulation in the myocardium, leading to hypertrophy [46]. Lipids on exosomes are essential to maintain their structural integrity and to transport their cargo into recipient cells. It is well established that lipids such as sphingomyelin or ceramide are crucial for the ceramide-dependent budding of intraluminal vesicles into MVBs. Inhibition of sphingomyelinases using GW4869 significantly reduced exosome production by cells [47]. This mechanism of exosome production by endocytosis of lipid rafts has also been confirmed in iPSC-derived MSCs by pulsing them with transferrin and cholera toxin B ligands [48]. The GW4869 sphingomyelinase inhibitor decreased exosome production in iPSC-MSCs in a dose-dependent manner [48]. The lipid layer of exosomes protects proteins from degradation and further preserves their functionality to induce therapeutic benefits on target cells [49]. In addition, the lipid composition on exosomes allows lipophilic dye labeling, which helps trace exosomes in vitro and in vivo [50]. The several advantages of exosomes compared to their parent cells make them an ideal therapeutic tool to treat CVDs and other diseases.

## 3. Stem Cell Exosomes for Cardiac Repair

Newer regenerative medicine approaches have sought to use the paracrine and mircrine mechanisms of stem and progenitor cells via exosomes to treat CVDs. Exosomes from adult stem cells such as bone marrow MSC exosomes are known to carry procardiogenic miRNAs such as miRNA-29 and miRNA-24, which elicit cardioprotective effects in vitro on H9C2 cells and the induced MI rat model [51]. MSCs from bone marrow, adipose tissue, and endometrium were all shown to be proangiogenic and improved cardiac function in a rat model of MI; however, endometrial MSC exosomes were enriched in miRNA-21, which specifically inhibited PTEN, increased Akt and VEGF in cardiac cells, and subsequently enhancing cardiac tissue viability [52]. In addition to MSCs, exosomes derived from cardiac progenitors have also gained momentum in repairing the injured heart tissue. For example, exosomes of CPCs from atrial appendage explants enriched with miRNA-210, miRNA-132, and miRNA-146a-3p inhibited apoptosis in HL-1 mice CMs [53]. In the study, CPC exosomes prevented cardiomyocyte apoptosis in MI hearts of rats mediated by miRNA-210 downregulation of ephrinA3 and PTP1b [53]. The CPC exosomes also entered into the endothelium of infarcted rat hearts and promoted angiogenesis by downregulating RasGAP-p120 mediated by miRNA-132 [53]. Another study showed that culturing human CPCs in a physiological oxygen concentration (5%) enhances the angiogenic activity of bovine aortic endothelial cells in vitro, and this was attributed to the paracrine function of endothelin-1 (EDN1) in the exosomes [54]. Although exosomes and their cargo components from adult stem cells improve cardiac function and promote angiogenesis in several animal models of MI, the complete reversal of cardiac function was not observed in most if not all studies [55].

Clinically, only one therapeutic study is actively recruiting patients with atrial fibrillation to be treated with epicardial fat pad exosomes (NCT034784010). Another clinical research study is also registered to treat 30 patients who have undergone aortic dissection using MSC exosomes along with a triple branched stent graft (NCT04356300). However, the type of MSCs from which the exosomes would be derived is not described, and recruitment has not begun. Several other registered clinical studies use the analysis of exosomes from urine or peripheral plasma as a diagnostic tool for CVDs (Table 1).

It is well established that neonatal mice or rats can regenerate their hearts after MI or apical resection, and the primary mechanism is by promoting cardiomyocyte proliferation [56]. Therefore, there may be a need for an in-depth analysis of signaling messages carried by exosomes derived from embryonic or neonatal-like PSCs to modify adult myocardial cells phenotypically and genetically into a temporary young phenotype to regenerate cardiac cells.

In the following sections of this review, we discuss stem cell-derived exosomes and their therapeutic value in cardiac regeneration.

### Pluripotent Stem Cell-Derived Progenitor Exosomes

Both ESCs and iPSCs are self-renewing, possess unlimited proliferation capacity, and have the ability to differentiate into endoderm, ectoderm, and mesoderm cells [57,58,59]. However, ethical concerns associated with obtaining embryos to produce ESCs are a significant setback [60]. PSCs are known to induce teratocarcinoma formation when transplanted into animals, in addition to the risks associated with genomic integration of viral and other vectors used for pluripotency reprogramming [61]. In contrast to cells, exosomes derived from PSCs are unlikely to induce teratoma formation in host tissues when transplanted [13].

Previous studies have established that microvesicles from ESCs with cargo enriched with mRNA coding for pluripotency (Oct-4, Nanog, Rex-1) and stem cell markers (Scl, HoxB4, GATA-2) promote the survival and expansion of hematopoietic progenitor cells, confirming that microparticles or other EVs are capable of inducing functional effects on differential cell types [62]. Comparably, exosomes from undifferentiated ESCs were shown to enhance cardiac function and angiogenesis and promote the survival and proliferation of cardiac progenitor cells (CPCs) in the ischemic myocardium of mice [12]. The recovery of infarcted mice hearts was associated with the role of miRNA-294 transferred from ESCs to CPCs via exosomes [12]. Similarly, EVs derived from undifferentiated iPSCs were enriched with miRNA-19b, miRNA-20s, miRNA-126-3p, miRNA-130a-3p, miRNA-210-3p, and miRNAs-19-27, also known as the “longevimiR cluster” [13]. Gene ontology analysis of these miRNAs revealed their specific roles in regulating cell proliferation, apoptosis, differentiation, self-renewal, and maintenance of pluripotency. Direct efficacy comparison of intramyocardially injected undifferentiated iPSCs and their EVs in a mouse model of ischemia-reperfusion injury revealed that iPSC EVs were significantly superior to their parent cells in reducing LV end-systolic volume and LV mass [13]. These studies underpin the fact that PSC-derived EVs or exosomes are likely to be equal or superiorly efficacious in treating CVDs than their parent cells. Unfortunately, only a few studies to date have evaluated the therapeutic efficacy of EVs or exosomes derived from undifferentiated PSCs.

Current literature evidence predicates that PSCs committed to the cardiac lineage are likely to improve cardiac function compared to adult stem cells. One study showed that although both MSCs and human iPSC-derived CMs were efficacious in MI-induced athymic nude rats, iPSC CMs were superior in reducing cardiac fibrosis and improving cardiac function [63]. In a similar model of rat MI, another study showed the greater efficacy of hydrogel-encapsulated iPSC-derived CMs’ enhanced cardiac function and increased infarct muscle content compared to hydrogel embedded erythropoietin [64]. Although the authors used advanced hydrogels to enhance the engraftment of iPSC-CMs, they did not find any cells by the end of 10 weeks [64]. The authors speculated that the improvement in cardiac function could be from the intracellular composition of dead cells. However, it is possible that the CMs exhibited paracrine factors, including microparticles and exosomes, which likely diffused into the ischemic myocardium of rats. Another study showed that iPSC-CM-derived EVs (iCM-EVs) enriched with cardiac-specific miRNAs such as miR1, miR99a, miR133a, miR143, miR145, and miR375 resulted in significantly reduced variance of instantaneous beating in hypoxic iCMs compared to iPSC-EVs [65]. The embedding of these iCM-EVs in hydrogels and implanting them in MI-induced athymic nude rats allowed the sustainable release of EVs, significantly reducing the overall arrhythmic burden and improving cardiac contractility compared to hydrogel-embedded iPSC-EVs [65]. Santoso MR et al. demonstrated that iPSC-derived CM exosomes promoted the survival of hypoxic CMs by increasing autophagic flux in addition to inhibition of mTOR signaling in the peri-infarct region of the heart [14].

A few scientific groups predicted that progenitor cells derived from embryonic or induced PSCs are likely to secrete exosomes with higher cardioprotective and regenerative potential compared to EVs or exosomes from more mature phenotypes derived from PSCs. Supporting this hypothesis, Kervadec A et al. showed that hESC-derived CPC-EVs significantly reduce LV end-systolic volume and induce a significant change in LVEF compared to their parent cells in a mouse model of chronic cardiac insufficiency [15]. The functional benefits caused by the PSC-derived CPCs and their EVs were associated with the upregulation of 927 genes involved in crucial biological processes such as cell survival, DNA repair, cell-cycle induction, and cardiomyocyte contractility. Importantly, this study reiterated that EVs or exosomes from PSCs or PSC-derived progenitor cells mirror the efficacy associated with cells and thereby eliminate the need to inject the cells [15]. Later, the same group showed that iPSC-derived CPC exosomes were enriched in 16 conserved miRNA, known to promote cardiogenesis, cell migration, survival, and viability [66]. In the same study, administration of iPSC-derived CPC exosomes significantly decreased LV end-systolic and end-diastolic volumes, improving LVEF, and promoted cardiomyocyte proliferation, as evidenced by EdU detection [66]. In a recent study, Wu Q et al. differentiated hESCs into cardiovascular progenitor cells (CVPCs), cultured them in normoxia and hypoxia, and collected their differentially secreted EVs to evaluate their angiogenic and cardiogenic effects in vitro and in vivo [16]. Both normoxic and hypoxic CVPC-EVs promoted equal migration of HUVECs in vitro; however, hypoxic CVPC-EVs promoted significantly higher tube formation of HUVECs compared to normoxic CVPC-EVs. Further, the intramyocardial delivery of these exosomes into MI-induced mice improved post-MI cardiac function and reduced scar size. Injection of hypoxic CVPC-EVs significantly reduced TUNEL^+^ cells in the injured myocardium of mice compared to normoxic CVPC-EVs. Interestingly, the efficacy of both CVPC-EV types was attributed to their MALAT1 lncRNA enriched cargo, which was shown to inhibit miRNA-497 to promote cardiomyocyte protection and angiogenesis [16].

These studies provide evidence of exosomal efficacy in the ischemic myocardium. However, the specificity of PSCs exosomes in entering and eliciting reparative functions in each of the different types of cells in the diseased heart is still unknown. It is factual that both embryonic and induced PSCs differentiate into various kinds of heart cells, such as CMs, endothelial cells [67], smooth muscle cells [68], and epicardial cells, which further differentiate into cardiac stromal cells and cardiac fibroblasts in vitro [69]. In the in vitro stages of PSC-directed differentiation, there is a precursor or progenitor stage for every cell type during the differentiation process. These progenitor cell exosomes are likely to carry specific cargo and regenerate the terminally differentiated cell type, partially damaged in a disease environment. It is evident that progenitor cell exosomes preferentially induce functional effects in their relevant mature cell types. However, they may also render therapeutic impact by delivering their cargo into other cell types in the disease microenvironment.

**Figure 1:**
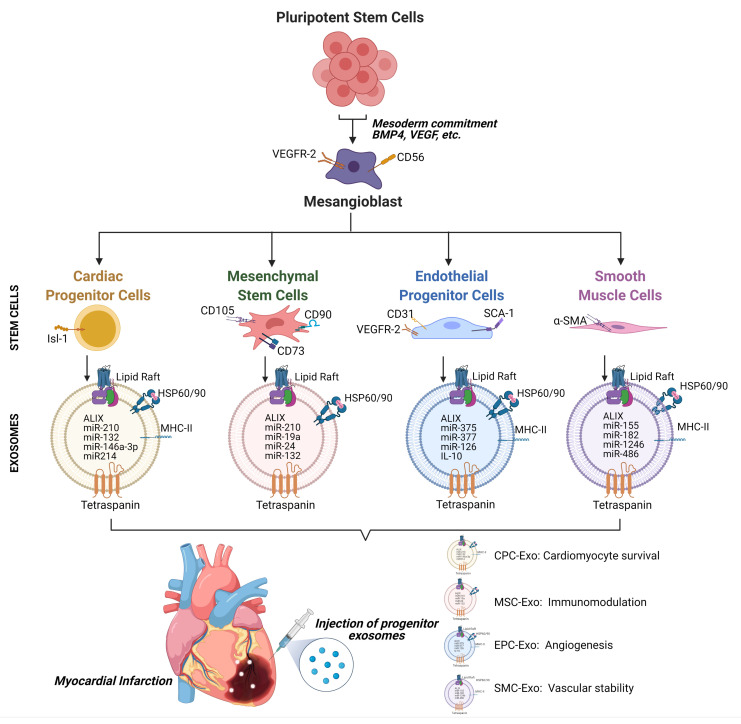
Generation of differential exosomes from various progenitor cells of the mesoderm lineage, derived from a common mesangioblast differentiated from PSCs for treating CVDs: PSCs can be cued to differentiate into the mesodermal lineage to form mesangioblasts expressing VEGFR2 and CD56. PSC-derived mesangioblasts could be further differentiated into Isl-1-expressing CPCs, MSCs expressing CD90, CD105, and CD73, CD31^+^ and Sca-1+ EPCs, and αSMA^+^ SMCs in vitro. These progenitor cells could be culture expanded on a large scale, and their exosomes can be collected. Therapeutic use of these exosomes in combination is likely to impart synergistic improvement in cardiomyocyte survival, the reduction of inflammation, and the induction of angiogenesis.

It is enticing to speculate that exosomes of a common progenitor or stem cell type made from PSCs in vitro could enter preferentially into all relevant mesodermal-origin cells in the damaged or ischemic myocardium and deliver nanomessages to induce repair and regeneration in the damaged heart. PSC-derived mesangioblasts expressing CD56 and low vascular endothelial growth factor receptor 2 (VEGFR2) are the known broad precursor cell type for cardiac cell types such as CMs, endothelial cells, MSCs, fibroblasts, pericytes, and smooth muscle cells. (Figure 1) [70]. PSC-mesangioblast exosomes are likely to carry reprogrammable cargo for mesodermal-origin cells in the myocardium. As discussed in the previous sections of this review, it is clear that exosomes from different progenitor cells such as CPCs, EPCs, and MSCs are therapeutically beneficial in repairing ischemic hearts by eliciting various mechanisms. However, evidence from many stem cells and exosome studies clearly show that complete or close-to-complete repair and regeneration of the myocardium has not been achieved so far. Incomplete repair is likely due to the variation in cargo between the cell types and the limited inherent regenerative ability of the adult myocardium. A recent systematic review found differential exosomal load in various cardiac cell types [71]. They found CM exosomes enriched in miRNA-217, miRNA-199, HSP90, and HSP60; cardiac endothelial cells enriched with miRNA-146a, miRNA-214, miRNA-143, miRNA-145; and cardiac fibroblasts containing miRNA-21-3p, miRNA-27a, miRNA-28-3p, and miRNA-34a, which regulate cardiac hypertrophy, fibrosis, and angiogenesis differentially [71]. Carefully selected exosome combinations from progenitor cells are likely to improve the magnitude of ischemic myocardium repair. In agreement with this hypothesis, the authors of a recently published intriguing study reprogrammed human cardiac fibroblasts into iPSC and then differentiated the iPSCs into CMs, endothelial cells (ECs), and smooth muscle cells (SMCs) [72]. The iPSC-derived cells were mixed at a ratio of 2:1:1 (CM:EC:SMC) and designated as iPSC-cardiac cells (iPSC-CC). These iPSC-CCs were further cultured to generate a mix of exosomes. These iPSC-CC exosomes were highly angiogenic in vitro and promoted angiogenesis, cell survival, and proliferation and enhanced the expression of promigratory CXCR4 in induced MI swine hearts as their parent iPSC-CC population. The mixed iPSC-CC exosomes were found to be safe and did not increase the risk of arrhythmias [72]. However, we cannot negate the possibility that exosomes from a single PSC-derived cell type could induce functional changes in many cell types. Correa BL et al. showed that iPSC-derived CPC exosomes are nonimmunogenic and seem to elicit anti-inflammatory responses by polarizing monocytes to an anti-inflammatory M2 phenotype [73]. Injection of iPSC-CPCs or their exosomes into the infarcted mouse hearts consistently reduced monocyte infiltration in the mouse hearts, and this was supported by reduced levels of pro-inflammatory cytokines IL-1α, IL-1β, IL-2, IL-6, TNFα, and IFNγ [73].

We hypothesize that the differentiation of PSC-mesangioblasts into Isl-1^+^ CPCs, Sca-1^+^CD31^+^ VEGFR2^+^ EPCs, CD73^+^ CD90^+^ CD105^+^ HLA-DR^-^ MSCs, and α-SMA^+^ smooth muscle cells (SMCs) followed by the collection of their exosomes comprising differential cargo could be beneficial in exploring combinatorial exosomal therapy for CVDs (Figure 1). The use of PSCs offers an unlimited supply of progenitor cells and their exosomes. Furthermore, unlike their parent stem cells, exosomes from partially differentiated PSCs are not known to induce tumorigenesis or teratogenesis in the target cells and tissues so far. In agreement with the previous statement, Adamiak et al. presented a study that demonstrated superior perfusion in the infarct zone rendered by iPSC-EVs compared to their parent cells in a murine model of MI, and more importantly, iPSC-EV administration did not lead to teratoma formation as their parent iPSCs [13].

The feasibility of expanding PSCs or their progenitors using good manufacturing practice (GMP)-grade serum-free, feeder-free systems on a large-scale is well established using several types of 3D bioreactors for further human therapeutic applications [74]. The validation of the expansion and scale-up of undifferentiated PSCs in a suspension culture system such as wave, rotating-wall, and stirred-tank bioreactors is well established [75]. An efficient way to generate CPCs is by first expanding undifferentiated PSCs in a suspension culture followed by their differentiation in an adherence system. The expansion of these CPCs could then be reconverted into a suspension culture while maintaining the phenotype and allowing their proliferation [76]. This or other similar processes will allow the collection of large volumes of media enriched with progenitor cell exosomes. The exosomes from the collected conditioned medium could then be collected using techniques such as immunoaffinity-based field-flow fractionation or by concentrating the conditioned medium using tangential flow filtration (TFF) followed by ultracentrifugation process [77]. Subjecting the conditioned medium to the latest techniques such as cyclic TFF systems [78] and further ultracentrifugation is likely to yield a high concentration of purified exosomes.

## 4. Discussion

The preferential entry of exosomes into the desired cell types in tissues or organs to elicit efficient repair and regeneration is unexplored. In the context of CVDs, the heart is an organ of mesoderm lineage and is likely to respond to exosomes from a common mesangioblast or progenitors differentiated from the mesangioblasts. Mesangioblasts can be derived from embryonic or induced PSCs and expanded on a large scale in vitro. Cardiovascular progenitor cells such as CPCs, EPCs, MSCs, and cardiac fibroblasts can be further derived from these multipotent mesangioblasts. Exosomes derived by the large-scale expansion of these progenitor cells used in experimentally determined combinations are likely to elicit an enhanced outcome compared to current strategies, which use exosomes from a single cell type. Although the cargo of different stem cell exosomes has been substantially characterized and evaluated in the context of CVDs, a large portion remains unfathomed. Further studies are required to fully understand the composition and functions of stem/progenitor cell exosomes.

## Figures and Tables

**Table 1:** Registered clinical trials using exosomes from different sources as a therapeutic or diagnostic tool.

Exosome Source	Disease Condition	Type	Clinical Trial Identifier
Epicardial fat pad exosomes	Atrial fibrillation	Therapeutic	NCT03478410; recruiting
MSC exosomes	Multi-organ failure due to aortic dissection	Therapeutic	NCT04356300; not yet recruiting
Urinary exosomes	Hypertension; detection of exosomal sodium; detection of exosomal Na^+^ channel proteins	Diagnostic	NCT03034265; completed
Urinary exosomes	Heart failure; autoimmune thyroid heart disease	Diagnostic	NCT04127591; not yet recruiting
Urinary exosomes	Heart failure; preserved ejection fraction	Diagnostic	NCT03837470; recruiting
Peripheral plasma	Myocardial infarction; miRNA expression analysis	Diagnostic	NCT03984006; recruiting
Peripheral plasma	Exosomal concentration with cardiopulmonary exercise	Diagnostic	NCT04334603; recruiting
Peripheral plasma	Vascular dysfunction associated with sleep apnea; inflammatory myeloid protein tyrosine phosphatase 1B (PTP1B) measurement	Diagnostic	NCT04235023; not yet recruiting
